# Cultural Adaptation of ADVANCED-Comfort for Chinese American Family Caregivers of Nursing Home Residents

**DOI:** 10.1093/geroni/igaf122.4346

**Published:** 2025-12-31

**Authors:** Andrea Wei, Benjamin Semple, Ruth Lopez

**Affiliations:** MGH Institute of Health Professions, Boston, Massachusetts, United States; Wake Forest University, Charlotte, North Carolina, United States; MGH Institute of Health Professions, Sharon, Massachusetts, United States

## Abstract

Family caregivers of nursing home (NH) residents with advanced dementia often prioritize comfort-focused care. However, culturally tailored tools to support this preference are lacking. This study aimed to culturally adapt the web-based ADVANCED-Comfort intervention and 6M Care Planning Tool to align with the values and caregiving practices of Chinese American families. We used a community-engaged, qualitative approach guided by principles of cultural tailoring and intervention mapping. A Chinese American Community Advisory Board (CAB) participated in a series of virtual meetings that included a concept mapping activity to define comfort and provide feedback on the intervention. Data were analyzed using qualitative description. CAB members identified five core themes of comfort: familiarity, respect, trust and emotional safety, connection to culture and family, and minimizing distress. These were richly illustrated with personal narratives, such as the importance of continuity in caregiving and culturally appropriate forms of address. A content mapping exercise showed strong alignment between these themes and the 6Ms of the intervention. Cultural adaptations included aesthetic refinements, incorporation of culturally specific imagery and symbolism, and greater emphasis on family naming and rituals. The 6M Care Planning Tool, co-completed by families and staff, generates a person-centered care plan that reflects shared values and is posted in the resident’s room. This cultural adaptation process demonstrated how participatory methods can enhance the relevance and acceptability of web-based interventions for diverse populations. The adapted ADVANCED-Comfort intervention is now better positioned to support culturally congruent, comfort-focused care for Chinese American families in NH settings.

